# Patient-reported use of pancreatic enzyme replacement treatment (PERT) in pancreatic cancer in New Zealand and Australia: a cross-sectional survey study

**DOI:** 10.1007/s00520-024-08604-1

**Published:** 2024-06-03

**Authors:** Amanda Landers, Helen Brown, Juhaina Al Ruheili, Kylie Russell, Clare McKenzie, Meera R. Agar, Vanessa M. Yenson, Kate Clarke, John Windsor

**Affiliations:** 1 Department of Medicine, University of Otago, 2 Riccarton Ave, Christchurch Central, Christchurch, 8011 New Zealand; 2 Nurse Maude Hospice Palliative Care Service, Nurse Maude Association, Christchurch, New Zealand; 3 Te Whatu Ora, Wellington, New Zealand; 4 Nutrition and Dietetics, Te Whatu Ora Te Toka Tumai, Auckland, New Zealand; 5 IMPACCT Centre, Faculty of Health, University of Technology Sydney, Sydney, Australia; 6 Surgical and Translational Research Centre, Faculty of Medical and Health Sciences, University of Auckland, Auckland, New Zealand

**Keywords:** Pancreatic cancer, Pancreatic exocrine insufficiency, Enzyme replacement therapy, Symptom management

## Abstract

**Purpose:**

This study investigated pancreatic enzyme replacement therapy (PERT) use in people diagnosed with pancreatic cancer in New Zealand (NZ) and Australia (AU).

**Methods:**

A cross-sectional survey study was conducted using a mixed-media campaign to recruit people with pancreatic cancer and collect information about current PERT use. The questionnaire gathered data on participant demographics, awareness of PERT, prescribing practices and efficacy of enzyme replacement.

**Results:**

Over 300 people with pancreatic cancer were recruited, 135 from New Zealand and 199 from Australia. Every region, state and territory was represented except for the West Coast (NZ) and the Northern Territory (AU), the lowest populated areas in both countries. In New Zealand, 60% of participants had heard about PERT, compared to 69.3% in Australia. Dosing regimens were inconsistent in both countries, with 18% and 27% of participants being prescribed PERT considered best practice in New Zealand and Australia, respectively. Before PERT commencement, 70% of participants experienced symptoms of malabsorption, with all symptoms improving after therapy was established. The majority of participants were compliant with their medication.

**Conclusion:**

PERT use in pancreatic cancer in New Zealand and Australia was highly variable and not compliant with international guidelines in which PERT is recommended as standard therapy. Enzyme replacement is effective for improving the symptoms of malabsorption in patients with pancreatic cancer. Clinician education may be needed to help improve the use of PERT in people with pancreatic cancer.

## Introduction

Pancreatic cancer has an increasing prevalence in many countries, with a uniformly dismal prognosis [1]. In New Zealand and Australia, approximately 630 and 4500 new cases are diagnosed each year, respectively, with a 12% 5-year survival rate [2, 3]. Indigenous populations have worse outcomes, with Māori patients more likely to die than non-Māori, and similarly poor outcomes for Aboriginal and Torres Strait Islanders [3, 4].

The pancreas is responsible for both endocrine (hormone production) and exocrine processes (enzyme secretion) in the body. Pancreatic cancer disrupts the delivery of enzymes to the duodenum secondary to ductal obstruction and is one of the most common manifestations of this cancer [5, 6]. Pancreatic exocrine insufficiency (PEI) can lead to malabsorption characterised by symptoms such as steatorrhoea, abdominal pain, weight loss, bloating and excess flatus [7].

Pancreatic enzyme replacement has been shown to improve quality of life in those with unresectable pancreatic cancer [8]. It is an oral medication of porcine origin taken at mealtimes to mimic normal digestion. This medication is readily available and funded in New Zealand and Australia, is well tolerated by patients and can have a positive impact on survival [9–11]. PERT administration for PEI in this cancer population is recommended as standard treatment in international cancer guidelines [12, 13].

Evidence from around the world suggests that in patients with pancreatic cancer, there is often a lack of assessment for pancreatic exocrine insufficiency, and a failure to prescribe PERT [14, 15]. The literature suggests variations in how PERT is prescribed by clinicians, particularly related to the dosage and timing [16]. Education of patients regarding PERT appears inconsistent and inadequate, which may contribute to poor compliance rates [17]. Lack of awareness regarding PERT and the correct administration may lead to poor quality of life and potentially reduced survival rates in people with pancreatic cancer [10]. The aim of this study was to survey patients with pancreatic cancer in NZ and AU to investigate the awareness of PERT, how PERT is prescribed and how effective it is in relieving symptoms of malabsorption.

## Methods

### Study design

This online cross-sectional survey study was designed and conducted from November 2021 to July 2022. Ethical approval was obtained from the University of Otago Human Ethics Committee (H21/069). The target population was people in New Zealand and Australia with a diagnosis of pancreatic cancer. The questionnaire was created utilising the online survey platform Qualtrics and remained open during the data collection period. A mixed media strategy was used to reach recruits indirectly through clinicians and directly through media channels.

### Questionnaire design

The PERT questionnaire was developed by an Australasian multi-disciplinary team including dietitians, nurses, doctors and consumers and aimed at understanding the current prescribing practices of PERT. The survey questions collected demographic data, patient knowledge of PERT, dosage regimens, symptoms of malabsorption prior to and after commencing PERT, and adverse effects.

During questionnaire development, there was consultation with Māori groups (Indigenous population of New Zealand) to improve engagement and cultural safety. Consultation was not taken for Australian Indigenous groups. The questionnaire was piloted by members of the wider research group not directly involved with the study. Feedback from this group, which included a consumer, was incorporated into subsequent drafts.

### Recruitment strategy

A media strategy was developed for recruitment to complete the survey across New Zealand and Australia. The details of the recruitment campaign, effectiveness and cost utilisation have been published elsewhere [18]. A mixed media campaign involved the establishment of a social media presence on platforms including Facebook, Instagram, Twitter, LinkedIn and Google. A link to the survey was advertised, with the first page containing information about the study, with the option to consent and proceed to completing the survey. Relationships with consumer organisations such as the Māori Womens League (NZ), Whipple’s Warriors (AU) and PanKind (AU) boosted the dissemination of the survey link. Clinicians provided pamphlets to their patients with information about the study and a barcode linked to the survey.

The research group met after the survey had been open for six months. The team decided to leave the study open for another 3 months as participants were still engaging with the social media posts and the survey.

### Data analysis

The survey results were analysed using descriptive statistical methods to obtain overall percentages, mean scores, standard deviations and ranges. Comparisons between countries were performed when appropriate to highlight differences and pooled together for questions regarding symptoms.

## Results

### Participant demographics

The study recruited 135 respondents from New Zealand and 199 participants from Australia, although not all surveys were fully completed. Most participants were in the 60–69 age category for both New Zealand (44%) and Australia (37%) populations (Table 1). The majority of participants identified as female, and the predominant ethnicity from the New Zealand group were NZ Europeans (78%) or Māori (16%). This would be representative of the national population demographics. In the Australian group, the most common ethnicity was Australian (78%), followed by other (12%), and New Zealander (8%).

**Table 1:** Demographic characteristics of New Zealand (*n* = 109) and Australian (*n* = 179) participants

Age group (%)	New Zealand	Australia
20–29	4	0
30–39	2	2
40–49	11	5
50–59	17	25
60–69	44	37
70–79	21	27
80–89	1	5
90 +	1	0
Gender (%)		
Female	59	53
Male	41	47

Most participants in New Zealand were from the Canterbury (26%), Auckland (23%) and Wellington (10%) regions. The only region not represented was the West Coast, the least populated area of New Zealand. The majority of Australian respondents were from New South Wales (37%) and Queensland (30%). All territories/states were represented except for the ‘Northern Territory’, the most sparsely populated area of Australia.

**Fig. 1:**
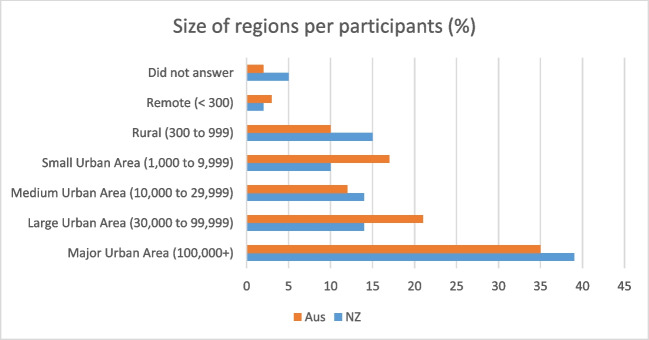
Size of regions per participants (percentage)

Most participants were from major urban areas (Fig. 1). This is consistent for both New Zealand (39%) and Australian (35%) populations. Smaller urban and rural populations were well represented in the population sample. Overall, there was a good distribution of participants from various regions including rural and smaller urban areas.

### Participant outcomes

Of the recruits from New Zealand, 51.4% of participants had not received surgery for their pancreatic cancer at the time of the study and of this sub-group 55.4% stated they were not going to have surgery. Rates of surgery were higher for the Australian population, with a smaller proportion (30.7%) not having had surgery and, 61.8% of this sub-group not planned for surgery.

Weight loss was experienced by most patients in both populations, with 65.1% of New Zealand participants stating they had lost weight since their diagnosis. From those experiencing weight loss, the most common amount of weight lost was 6–10 kg (40%), 11–15 kg (25%) and 16–20 kg (25%). A higher proportion of the Australian group experienced weight loss (77.65%). The most common amount reported being lost was 6–10 kg (26.6%), 20 kg + (25.9%) and 11–15 kg (22.3%). The majority of participants in both New Zealand (60.2%) and Australian (69.2%) populations had seen a dietitian.

### PERT awareness

Approximately 61% of participants (*n* = 66) in New Zealand had heard of PERT, with the most common sources of education about enzyme replacement from surgeons (39%), oncologists (20%) and dietitians (20%). Of the Australian participants, 69.3% (*n* = 124) had heard of PERT. Consistent with the New Zealand results, most had heard of PERT from their surgeon (50%), oncologist (20%) and dietitian (12%).

### PERT utilisation

For both groups, approximately 80% of clinicians had explained to patients how PERT works and 93% of patients been told when to take PERT by the clinician. PERT explanation rates from pharmacists were lower, with around 30% of participants from both countries receiving an explanation from the pharmacist who dispensed their PERT. Furthermore, only 47% and 41% of New Zealand and Australian pharmacists, respectively, explained to their patients when to take PERT. For both groups, only 53% of participants received written information. Few participants were directed to localised and culturally appropriate resources on PERT.

**Fig. 2:**
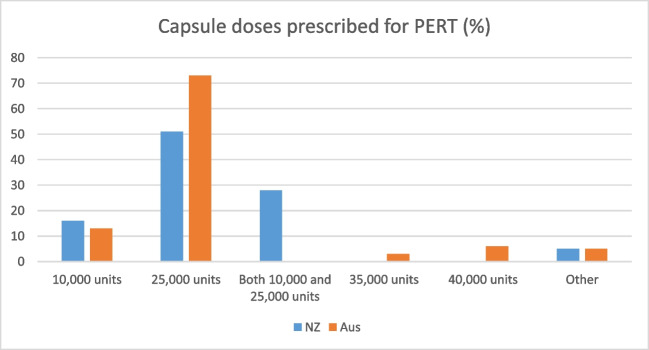
Capsule doses prescribed for PERT (%) in New Zealand (*n* = 66) and Australian (*n* = 124) populations taking PERT

The most common capsule dose provided for New Zealand (51%) and Australian (73%) populations was the 25,000-unit capsule (Fig. 2).

**Fig. 3:**
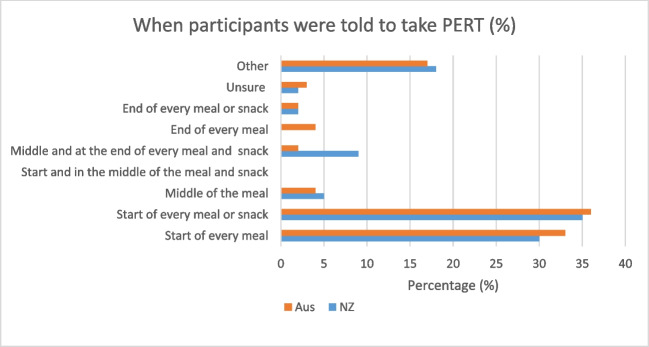
Time patients were recommended to take PERT relative to their meal

Among the New Zealand population, most (25%) participants were told to adjust the dose themselves depending on what they were eating. Following this, the most common recommendations were to take ‘two capsules with every meal and one with every snack’ (18%) and to take ‘two capsules with every meal’ (14%) (Fig. 3). In comparison, among the Australian population, the most common recommendations were to take ‘two capsules with every meal and one with every snack’ (27%), ‘one capsule with every meal’ (19%) and ‘other’ (11%). When inquired how often they took PERT, participants seem to be adherent to the medication. In total, 89% of New Zealand participants, and 75.9% of Australian participants stated they either took PERT ‘with all meals and snacks’, ‘with all meals, snacks and fluids (not water)’ or ‘each time I ate’. For the Australian results, 15.5% of participants selected the ‘other’ category.

Among the New Zealand participants, 10% reported barriers to medication, lower than the 25% of participants from Australia who experienced barriers to accessing PERT. The most common barrier was lack of availability at the pharmacy (46.4%).

### PERT and symptoms

**Fig. 4:**
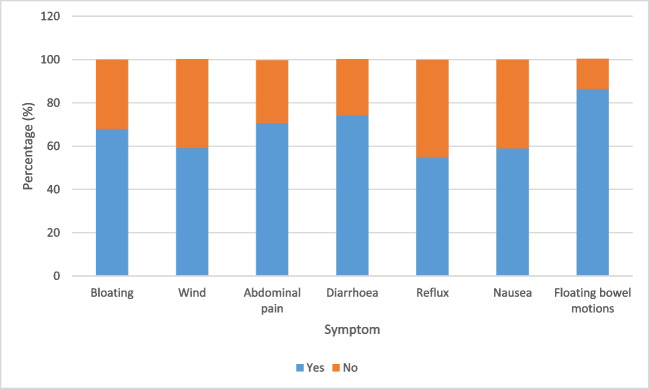
Symptom improvement for New Zealand (*n* = 34) and Australian participants (*n* = 89) after starting PERT

PERT use was consistently associated with an improvement in symptoms (Fig. 4). Most benefits were reported in improving floating bowel motions (86%), diarrhoea (74%) and abdominal pain (71%). In the New Zealand population, 11% of participants on PERT experienced side effects such as bloating, pain and dry mouth. This was higher in the Australian cohort (22%) and included headaches, nausea and wind.

## Discussion

This cross-sectional mixed media survey study captured a range of participants with pancreatic cancer across New Zealand and Australia. Our study engaged with people diagnosed with pancreatic cancer directly and asked them to report on their experience of PERT. Patient-reported outcome studies encourage patients to identify their symptoms, help determine the efficacy of medications and can prompt action [19]. Our study highlighted the lack of awareness of PERT in New Zealand and Australian participants with pancreatic cancer, with 30–40% of those respectively reporting they had not heard of PERT. There were highly variably prescribing patterns that are inconsistent with international guidelines, despite PERT being found to be highly effective in improving malabsorption symptoms. Compliance with PERT was found to be high amongst the participants once started on the medication.

Evidence from across the world has shown that PERT is under-utilised as the intervention for PEI in pancreatic cancer patients [15, 20, 21]. A recent national prospective study in the UK reported significant variation in the prescription of PERT between those with resectable disease and metastatic diagnoses [21]. In this study, a smaller proportion of patients were on PERT if they had unresectable disease, treatment at a regional centre, were older and had multiple co-morbidities. A US study, which used a patient registry disseminated through a patient advocacy group to investigate lived experience with PERT and pancreatic cancer, reported 85% of participants had spoken to a health professional about PERT, with 89% of them prescribed PERT [22]. The majority of prescribers were medical oncologists, in contrast to our study which demonstrated surgeons as the most common prescriber. Carnie et al. assessed the feasibility of an algorithm to guide prescribing of PERT and showed that almost 88% of participants were on PERT, although almost 50% were taking it incorrectly [16]. They also reported that compliance with the treatment was only moderate. Our results in this study showed the majority of people on PERT took the medication as prescribed. Current guidelines state that the best-practice dosing regime is to have a starting dose of two capsules with every meal and one with every snack, with up titration for large or high fat meals and snacks [23]. Only 18% and 27% of the participants in New Zealand and Australia were reported to have received this instruction in our study, with a wide variety of advice given to patients in both countries around dosing regimen. Patients, therefore, followed advice and prescription instructions that were not best practice.

Evidence from previous studies suggests that PERT plays a major role in symptom management, and potentially survival, for people with pancreatic cancer [10, 16, 22]. A US study reported symptoms in 88% of participants with pancreatic cancer, with self-reported resolution of these symptoms in almost half the recruits [22]. A 2023 Italian retrospective study of patients undergoing first-line chemotherapy on PERT reported less weight loss, improved tolerance of chemotherapy and significantly improved overall survival [24]. Our study outcomes are consistent with current literature, supporting the use of PERT to improve symptoms such as abdominal pain, bloating and diarrhoea which are known to significantly impact on the quality of life of people with pancreatic cancer [8]. Few adverse effects from the use of PERT in pancreatic cancer have been reported, suggesting the medication is safe and well-tolerated [9]. Our study found similarly low rates of side effects from enzyme replacement that could be attributable to the medication.

### Implications for clinical practice

There are a significant number of patients in New Zealand and Australia with pancreatic cancer who are likely to have malabsorption secondary to PEI and are not receiving PERT as a standard of care. Our study has demonstrated that patients are often prescribed an incorrect dose and/or timing regimes. Patients experienced few adverse effects related to the enzyme replacement. We therefore recommend that clinicians should be discussing PERT with all patients diagnosed with pancreatic cancer, which is consistent with international guidelines.

### Strengths and limitations

This survey study has some limitations. As it was completed by patients without any assistance from a clinician, the diagnosis was self-reported. The survey was advertised through mixed media and would only target those with access to platforms such as Instagram, Facebook and Twitter. This methodology may not have engaged participants from culturally and linguistically diverse populations, homeless or people from lower socio-economic backgrounds. The main strength of this study was effective promotion of the study to potential participants and improved recruitment efforts, especially in rural areas. Our research team also had strong connections with multiple consumer advocacy groups for people with pancreatic cancer.

## Conclusions

Pancreatic cancer is often associated with PEI and malabsorption which reduces quality of life. PERT is a simple, well-tolerated and effective treatment for PEI that not only improves symptoms but can also prolong survival. In New Zealand and Australia, it has been found that 30–40% of patients are unaware of PERT, and when prescribed, is done in a high variable way. There is a need for clinician education to ensure that all patients with pancreatic cancer are considered for PERT as a standard of care. An implementation randomised controlled trial of PERT in pancreatic cancer would help understand how to increase awareness amongst patients and clinicians and is in the planning phase by our research group.

## Data Availability

Data from this study is available on request from the authors.
